# The Fungi-specific histone Acetyltransferase Rtt109 mediates morphogenesis, Aflatoxin synthesis and pathogenicity in *Aspergillus flavus* by acetylating H3K9

**DOI:** 10.1186/s43008-021-00060-4

**Published:** 2021-04-07

**Authors:** Ruilin Sun, Meifang Wen, Lianghuan Wu, Huahui Lan, Jun Yuan, Shihua Wang

**Affiliations:** Key Laboratory of Pathogenic Fungi and Mycotoxins of Fujian Province, Key Laboratory of Biopesticide and Chemical Biology of Education Ministry, and College of Life Sciences, Fujian Agriculture and Forestry University, Fuzhou, 350002 China

**Keywords:** *Aspergillus flavus*, Histone, Acetylation, *rtt109*, H3K9

## Abstract

*Aspergillus flavus* is a common saprophytic filamentous fungus that produces the highly toxic natural compound aflatoxin during its growth process. Synthesis of the aflatoxins, which can contaminate food crops causing huge losses to the agricultural economy, is often regulated by epigenetic modification, such as the histone acetyltransferase. In this study, we used *Aspergillus flavus* as an experimental model to construct the acetyltransferase gene *rtt109* knockout strain (△*rtt109*) and its complementary strain (△*rtt109*·com) by homologous recombination. The growth of △*rtt109* was significantly suppressed compared to the wild type (WT) strain and the △*rtt109*·com strain. The sclerotium of △*rtt109* grew smaller, and the amount of sclerotia generated by △*rtt109* was significantly reduced. The number of conidiums of △*rtt109* was significantly reduced, especially on the yeast extract sucrose (YES) solid medium. The amount of aflatoxins synthesized by △*rtt109* in the PDB liquid medium was significantly decreased We also found that the △*rtt109* strain was extremely sensitive to DNA damage stress. Through the maize seed infection experiment, we found that the growth of △*rtt109* on the surface of affected corn was largely reduced, and the amount of aerial mycelium decreased significantly, which was consistent with the results on the artificial medium. We further found that H3K9 was the acetylated target of Rtt109 in *A. flavus*. In conclusion, Rtt109 participated in the growth, conidium formation, sclerotia generation, aflatoxin synthesis, environmental stress response, regulation of infection of *A. flavus.* The results from this study of *rtt109* showed data for acetylation in the regulation of life processes and provided a new thought regarding the prevention and control of *A. flavus* hazards.

**Supplementary Information:**

The online version contains supplementary material available at 10.1186/s43008-021-00060-4.

## Introduction

In eukaryotic organisms, chromatin is a dynamic and highly regulated structure that functions in compacting DNA into the nucleus and affects vital functions, such as gene expression, cell differentiation, and cell division. The fundamental repeat unit of chromatin is the nucleosome, which is composed of two copies of histone proteins H2A, H2B, H3, and H4, and wrapped twice around by about 147 base pairs of circumvoluted DNA. Histone modifications are understood to have the capacity of regulating transcription apart from genome expression (Kornberg & Lorch, 1999; Strahl & Allis, 2000). Some nuclear activities are strongly affected by different posttranslational modifications to the residues of histone proteins, including acetylation, methylation, phosphorylation, SUMOylation and ubiquitination (Hamon et al., 2007; Arbibe et al., 2007).

In the case of acetylation, it is a reversible process mediated by both histone acetyltransferases (HATs) and histone deacetylases (HDACs), by which a range of functions operate. HATs interacting with acetyl-coenzyme A, a co-factor that transfers an acetyl-group to a lysine residue, allows chromatin to transform into a flexible structure, and thereby activate transcription (Shahbazian & Grunstein, 2007). In contrast, HDACs deacetylate acetylated-lysine residues, compress DNA sequences, and lead to transcription repression (Kouzarides, 2000). Generally, HATs are grouped into 13 families according to their evolutionary relationships, where each HAT shares similarities with the others and are thus named from KAT1 to KAT13 (Torchia et al., 1998).

There is more evidence implicating that the acetylation on histone residues plays an important role for many cellular physiological processes in eukaryotes. For example, Gcn5, a KAT2-family HAT (Carrozza et al., 2003), was reported to participate in the regulation of nearly 12% of the genome in yeast (Lee et al., 2000). Deletion of KAT5-family HAT Esa1, led to lethality in *A. nidulans (Soukup et al.,* 2012*)*. In spite of the acetylation of histone and the functions of relative HATs have been studied extensively, there are still many functions that have not been fully characterized in fungi.

The regulator of Ty1 transposition 109 (Rtt109) was identified as a fungal-specific KAT11-family histone acetyltransferase, mainly responsible for the acetylation of histone 3 lysine 56 (H3K56) (Carrozza et al., 2003). This regulator was also reported to have a slight relationship with the acetylation of several residues on H3K9 and H3K27 (Ransom et al., 2010; Han et al., 2007a; Driscoll et al., 2007; Tsubota et al., 2007a; Berndsen et al., 2008; Schneider et al., 2006). The H3-H4 histone chaperone Asf1 primarily integrates to H3, and following that, H3K56 undergoes acetylation by Rtt109 (Tsubota et al., 2007b; Selth & Svejstrup, 2007a). A deletion mutant of Asf1 resulted in a large decrease to the level of acetylation on H3K56 (Han et al., 2007b). Another chaperone that has a relationship with Rtt109 is Vps75, a member of the Nucleosome Assembly Protein (NAP-1) family first found in *Saccharomyces cerevisiae* (Tang et al., 2008). In light of the evidence that Vps75 could combine with Rtt109 to form a Rtt109-Vps75 complex, influencing the level of acetylation on H3K56, Vps75 was found not essential for the acetylation of H3K56 (Selth et al., 2009; Selth & Svejstrup, 2007b; Park et al., 2008; da Rosa et al., 2013). The acetylation of H3K56 is vital for DNA damage repair, implying that the activation of DNA metabolic functions was facilitated by acetylation on H3K56 in *Saccharomyces cerevisiae *(Kadyk & Hartwell, 1992). Increasing evidence has demonstrated that cells would show a certain degree of inhibition under the pressure of DNA toxin agents without the acetylation on H3K56 in eukaryotic cells (Johnson & Jasin, 2000; Gonzalez-Barrera et al., 2003; Cortes-Ledesma & Aguilera, 2006; Gunjan et al., 2005).

Filamentous fungi are regarded as a class of eukaryote that can produce many kinds of bioactive compounds, known as secondary metabolites, which provide a selective advantage for themselves in the living environment and can be extremely harmful to other species (Yu & Keller, 2005). Many compounds are synthesized and released when fungi invade plants or animals, causing poisoning, diseases, and even death (Yu & Keller, 2005; Amaike & Keller, 2011; Amare & Keller, 2014). *Aspergillus flavus* is one kind of typical pathogenic filamentous fungus homologous to the well-established model organism *A. nidulans* (Zhang et al., 2015). It is also known as a common pathogen colonizing crops or food such as maize, peanuts, and rice (Gunjan et al., 2005; Yu & Keller, 2005; Amaike & Keller, 2011; Amare & Keller, 2014). *A. flavus* can produce aflatoxins resulting in billions of dollars of economic losses and serious food safety worldwide (Yu & Keller, 2005). Certain epigenetic factors could affect the growth, development and aflatoxin synthesis in *A. flavus*, such as methylation, acetylation, and phosphorylation. Many acetyltransferases including Rtt109 have been reported in fungal species, including *S. cerevisiae*, *A. fumigatus*, *A. nidulans*, and others (Amaike & Keller, 2011). However, the function of acetyltransferase Rtt109 has not been widely reported in *A. flavus*.

The purpose of this study is to clarify the effects of histone acetylation modification in growth and development, metabolism regulation, and infection of *A. flavus*. This can be used for which can be used for one of the targets for the control of aflatoxin in the future, and to lay a theoretical foundation for the prevention and control of aflatoxin. In this study, we studied the function of acetyltransferase Rtt109 in *A. flavus.* We found that Rtt109 in this fungus participated in growth, conidium formation, sclerotia generation, aflatoxin synthesis, environmental stress responses, regulation of infection, and other life processes. We further showed that many of those functions were influenced by the acetylation of H3K9, and revealed that H3K9 might be a candidate target for reducing both the fungal load and aflatoxin quantities in susceptible crops possibly through the application of HIGS (host-induced gene silencing).

## Materials and methods

### Sequence and phylogenetic tree analysis

To acquire an *A. flavus* Rtt109 protein sequence, a BLASTp search for protein homologs of the *S. cerevisiae* Rtt109 protein was conducted, and sequences of interest were downloaded from the National Center for Biotechnology Information resources (NCBI). These sequences were aligned with the ClustalW method, using MEGA 5.1 software, and a neighbor-joining phylogenetic tree was constructed according to the results. The visualized domains of Rtt109 or Rtt109-like proteins were constructed with DOG 2.0 software accordingly (Ren et al., 2009).

### Mutant strains construction

The fungal strains used in this study are listed in Table S1 and were constructed using the primers listed in Table S2. All strains were constructed via homologous recombination and grown on five plates (Zhang et al., 2015). The strategies for strain construction are shown in Fig. S1. For constructing the *rtt109* gene-deletion mutant (Δ*rtt109*), an overlap polymerase chain reaction (PCR) method was used as previously described (Chang et al., 2010; Szewczyk et al., 2006), replacing the original *rtt109* gene in *A. flavus* with the *pyrG* gene as a selection marker (Fig. S1A). For constructing the *rtt109* complementary strain (△*rtt109*·com), the sequence, which consisted of *rtt109* connected with *pyrG* and the overlaps, were used to replace the *pyrG* in Δ*rtt109* (Fig. S1B). For the gene-fluorescent fusion protein strain construction (*rtt109*-mCherry), the original sequence of *rtt109* were mutated into the one lacking a termination codon (TAG), and then joined to the gene sequence of a fluorescent protein (mCherry) (Wong et al., 2008a) followed by the *pyrG* sequence (Fig. S1C). The point mutant strains were established by an overlap PCR method to construct a sequence, of which the *pyrG* marker was downstream of the gene coding histone 3. The histone 3 gene sequence was mutated by PCR amplification using different modified PCR primers, changing the lysines into alanine, arginine, and glutamine, and the H3K9R, H3K9A, and H3K9Q strains were constructed, respectively (Fig. S2).

### Microscopic examination of the rtt109-mCherry subcellular localization

To assess the *rtt109*-mCherry localization, the newly formed fresh conidia and mycelia were observed using a Leica Confocal SP8 microscope system (Wong et al., 2008b). To observe the localization of the nuclei in mycelia, the samples were stained with 1 μg/mL 40, 6-diamidino-2-phenylindole (DAPI, Sigma, USA) for 30 min on ice. The samples were stained with 1 μg/mL 7-amino-4-chloroMethlycoumarin (CMAC, Sigma, USA) for 1 h at 37 °C, and then cultured in YGT liquid media at 37 °C for 2 h before observing.

### Western blot analysis of histone modification

Similar procedures were conducted in our previous work (Lan et al., 2016). The strains were incubated at 28 °C for 72 h, and the mycelia of each sample were collected, then frozen in liquid nitrogen and ground into powder. Next, 100 mg powder was dissolved in 1 mL radio immuno precipitation assay lysis buffer (RIPA, Beyotime, Shanghai, China), which was used to extract the whole proteins. The concentrations of the protein extracts were measured using a Nanodrop detector and then diluted. We uploaded 40 μg of total protein and separated them by 15% sodium dodecyl sulfate polyacrylamide gel electrophoresis. The proteins were then transferred to a polyvinylidene fluoride membrane (Millipore, USA). We used the specific antibodies anti-actin (1:5000 dilution, Cell Signaling Technology), anti-Histone3 (1:5000 dilution, Abcam), anti-acetyl-histone3 (H3ac, 1:5000 dilution, Abcam), anti-acetyl-H3K56 (H3K56ac, 1:5000 dilution, Active Motif), and anti-acetyl-H3K9 (H3K56ac, 1:5000 dilution, PTM Biolabs) to detect histone modifications.

### Analyses of conidia and Sclerotia

Conidia of each strain was removed from the periphery of a 2-day-old colony growing on yeast extract sucrose (YES) solid medium (Yang et al., 2016). The plugs were used to inoculate potato dextrose agar (PDA), YES, YS, and glucose minimal medium (GMM) (Shimizu & Keller, 2001). All strains were incubated at 37 °C without light, and the colony diameters were measured and recorded daily. After 5 days, conidia were collected in triplicate from 1 cm diameter zones of the agar media containing fungal growth. Samples were homogenized and diluted in 3 mL 0.05% Tween-20. Conidia were counted using a hemocytometer and microscope. To analyze the sclerotia, each strain was grown in Wickerham (WKM) agar medium at 37 °C in darkness (Raper & Thom, 1949). After 7 days, the surface of each plate was washed using 75% ethanol. Each strain was analyzed using five plates, and each experiment was repeated three times.

### Colonization assay

A colonization assay on maize seed was completed using a modified procedure (Kale et al., 2008). The spore solution, WT, △*rtt109*, △*rtt109*·com, H3-pyrG, H3K9R, H3K9Q, and H3K9A strains were diluted by water (approximately 10,000 spore/mL), and then the embryo-removed maize (*Zea mays*) seeds were added and cultured for 3 h at 28 °C with shaking (180 r/min). Maize seeds were harvested and transferred to a new plate with filter papers, and then cultured for 7 days. The filter papers were kept wet by adding 1 mL water every day.

### Determination of Aflatoxin B1 production

The aflatoxin B1 (AFB1) production was measured by thin-layer chromatography (TLC) and high performance liquid chromatography (HPLC) analysis (Yang et al., 2016). Briefly, strains were inoculated in 50 mL of liquid medium, and cultured at 29 °C for 72 h. The cultures were combined with 50 mL chloroform in 250 mL flasks and shaken for 30 min. The grown mycelia were collected and completely dried and weighed. Next, the organic residue of each sample was collected into a new plate, completely dried and weighed, and then resuspended in chloroform solvent according to the weight of the dried mycelia (1 μL/mg of mycelia) (Lan et al., 2016). Approximately 10 μL of samples were loaded onto TLC plates (Haiyang Chemical, Qingdao, China) and dried. Then, they were separated using the developing solvent (chloroform:acetone = 9:1) and exposed under UV radiation (365 nm). The TLC plates were photographed using a Quantum ST5 imaging system (Vilber Lourmat Deutschland GmbH, Eberhardzell, Germany). For HPLC analysis, the sample of aflatoxin extracts were analyzed using a Mycotox TM column (Waters, Milford, MA, USA) at 42 °C (Lan et al., 2016). AFB1 and AFB2, the main products of *A. flavus*, were identified using a fluorescent detector (Waters) (Lan et al., 2016).

### Stress assay

WT, △*rtt109*, △*rtt109*·com, H3-pyrG, H3K9R, H3K9Q, and H3K9A strains were used to inoculate PDA medium supplemented with the following agents: cell wall stress agent, Congo red (CR, 500 μg/mL); hyperosmotic stress mediator, sodium chloride (NaCl, 1.5 mmol/mL); genotoxic agent, methyl methanesulfonate (MMS, 0.01%, *v/v*); and oxidative stress agent, hydrogen peroxide (H_2_O_2_, 10 mmol/mL). The inoculated plates were incubated at 37 °C in darkness for 5 days. Colony diameters were measured every day. Each strain was cultured on five plates, and each experiment was repeated three times.

### Quantitative reverse transcription polymerase chain reaction

Mycelia were grown in YES liquid medium for 24 h, before being harvested and grounded into powder using liquid nitrogen. The total RNA was isolated from approximately 100 mg ground mycelia of each strain using an RNA isolation kit (Promega, USA). For qRT-PCR analysis, we used a Revert Aid First-strand cDNA Synthesis kit (Thermo Fisher Scientific, USA) to synthesize the cDNA from the RNA of each strain. We used the SYBR Green Supermix (Takara, Japan) and PikoReal 96 Real-time PCR system (Thermo Fisher Scientific, USA) to complete qRT-PCR analysis (denaturation at 95 °C for 30 S, renaturation and extension at 60 °C for 30 S, 40 cycles), and the relative quantities of each transcript were calculated using the 2^−△△CT^ method (Livak & Schmittgen, 2001). The transcript levels of qRT-PCR were normalized relative to the transcript level of the *β-tubulin* housekeeping gene. The qRT-PCR primers are listed in Table S3. The data of qRT-PCR analysis were completed in triplicate, and each experiment was repeated three times. In this study, we analyzed sclerotia production related gene *sclR*, and the aflatoxin synthesis related genes *aflO* and *aflR* to evaluate the acetylation effects on sclerotia production and aflatoxin synthesis respectively.

### Statistical analysis

Our data was presented as the means ± standard deviation of at least three biological replicate samples in figures. Statistical and significance analysis were performed using the data analysis software GraphPad Prism 5.1, and the *p*-values were lower than 0.05. As for multiple comparisons, we used Tukey’s multiple comparison for significance analysis.

## Results

### Identification of Rtt109 homologs protein in *A. flavus*

**Fig. 1:**
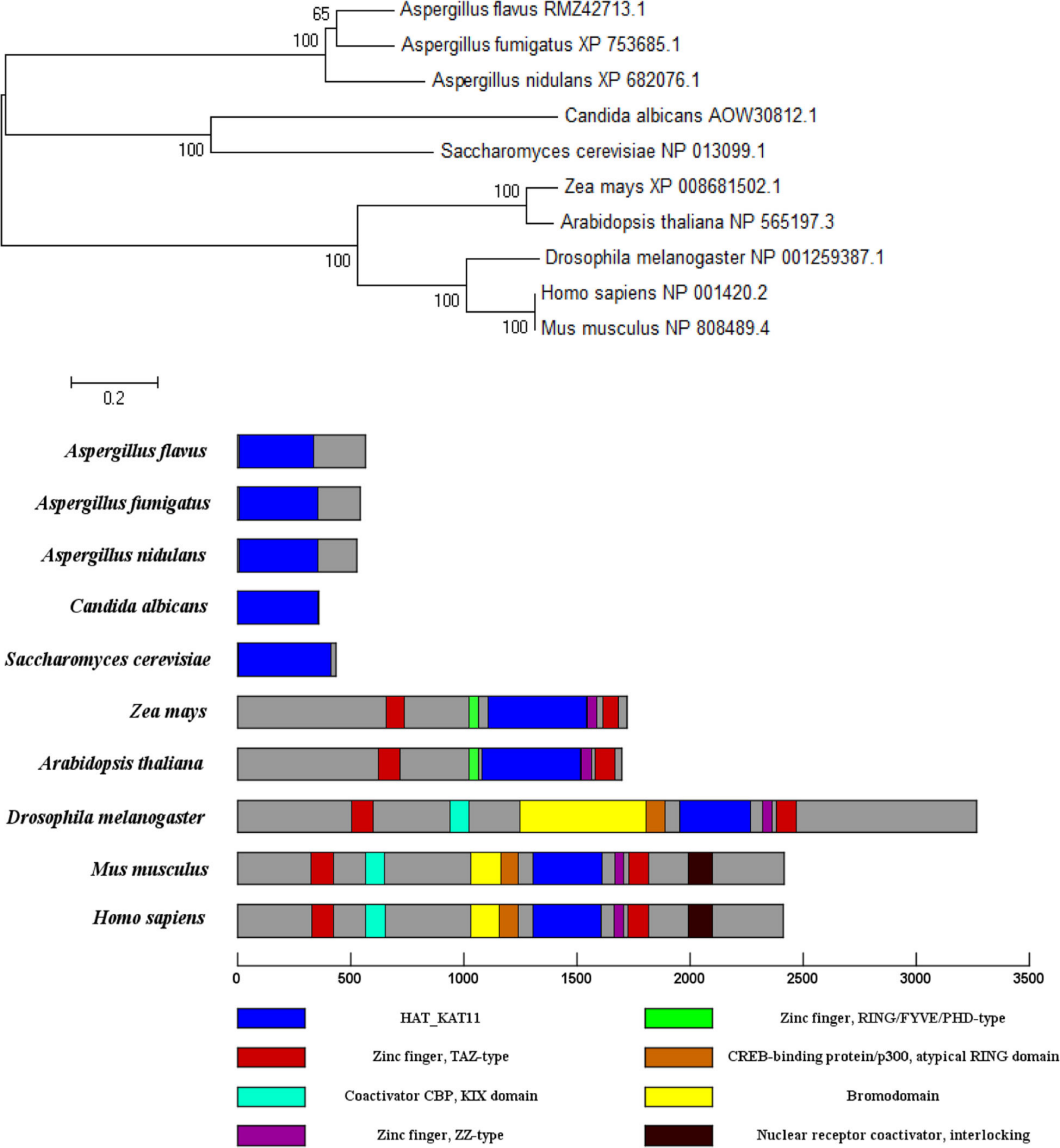
Our bioinformatics analysis of Rtt109 using the protein sequences of *A. flavus* (RMZ42713.1), *A. nidulans* (XP_682076.1), *A. fumigatus* (XP_753685.1), *C. albicans* (AOW30812.1), *S. cerevisiae* (NP_013099.1), *Z. mays* (XP_008681502.1), *A. thaliana* (NP_565197.3), *D. melanogaster* (NP_001259387.1), *M. musculus* (NP_808489.4)*,* and *H. sapiens* (NP_001420.2) for (**a**) phylogenetic trees*,* and (**b**) protein domain analysis

The DNA sequence of the putative DNA damage response protein Rtt109 in *A. flavus* was identified by searching the NCBI database. The *A. flavus rtt109* consisted of 1695 bp with only one intron, encoding a putative histone acetylase with 564 amino acids. A BLAST search and phylogenetic analysis revealed that Rtt109 or its homologous proteins were also found in *Saccharomyces cerevisiae*, *Aspergillus niger, Aspergillus flavus, Aspergillus fumigatus, Aspergillus nidulans, Zea mays, Arabidopsis thaliana, Dorsophilia melanogaster, Mus musculus,* and *Homo sapiens* (Fig. 1a). Despite that the lengths of amino acid sequence above were different, they shared the same protein domain KAT_11 and are highly conserved (Fig. 1b). These data suggested that Rtt109 proteins are conserved from fungi to higher eukaryotes.

### Rtt109 is a typical B-type HAT predominantly localized in vacuoles or vesicles

**Fig. 2:**
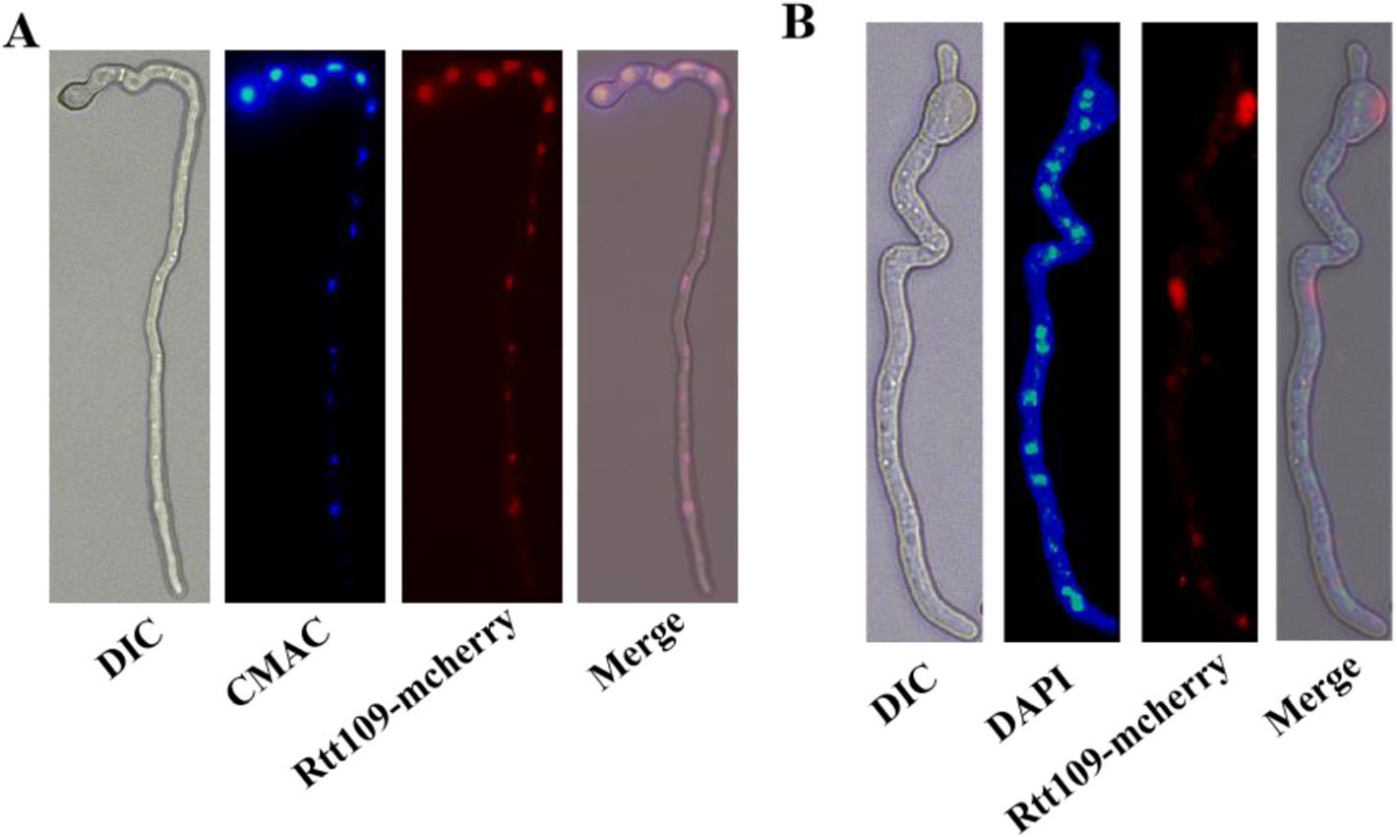
Subcellular localization of acetylase Rtt109 in *A. flavus.* The newborn mycelium of *rtt109*-mcherry strain was staining and observed under exciting light by using (**a**) 1 mg/mL 7-amino-4-chloroMethlycoumarin (CMAC) and (**b**) 1 mg/mL 6-diamidino-2-phenylindole (DAPI)

There are two types of intracellular HATs, including A-type HATs for acetyltransferases localized and functioning in the nucleus, and B-type HATs for acetyltransferases predominantly existing in intracytoplasmic regions, which are transported into the nucleus to function. In order to investigate the subcellular localization of the Rtt109 protein, we used a previously described method (Lan et al., 2016) to construct an *rtt109*-mCherry strain constitutively expressing the mCherry tag at the C-terminal of the *rtt109* sequence under the control of the native promoter in *A. flavus*. The *rtt109*-mCherry strain exhibited the same phenotype as the WT strains, suggesting that the function of *rtt109* was not affected by the mCherry tag. We observed that the rtt109 protein was primarily co-localized with a CMAC stained vacuole or vesicle in fluorescence microscopy (Fig. 2a), but not with a DAPI stained nucleus at the germination stage (Fig. 2b). These data suggest that Rtt109 is predominantly stored in vacuoles or vesicles typical of other B-type HATs.

### Rtt109 is a Bona fide HAT for acetylation of histone 3

**Fig. 3:**
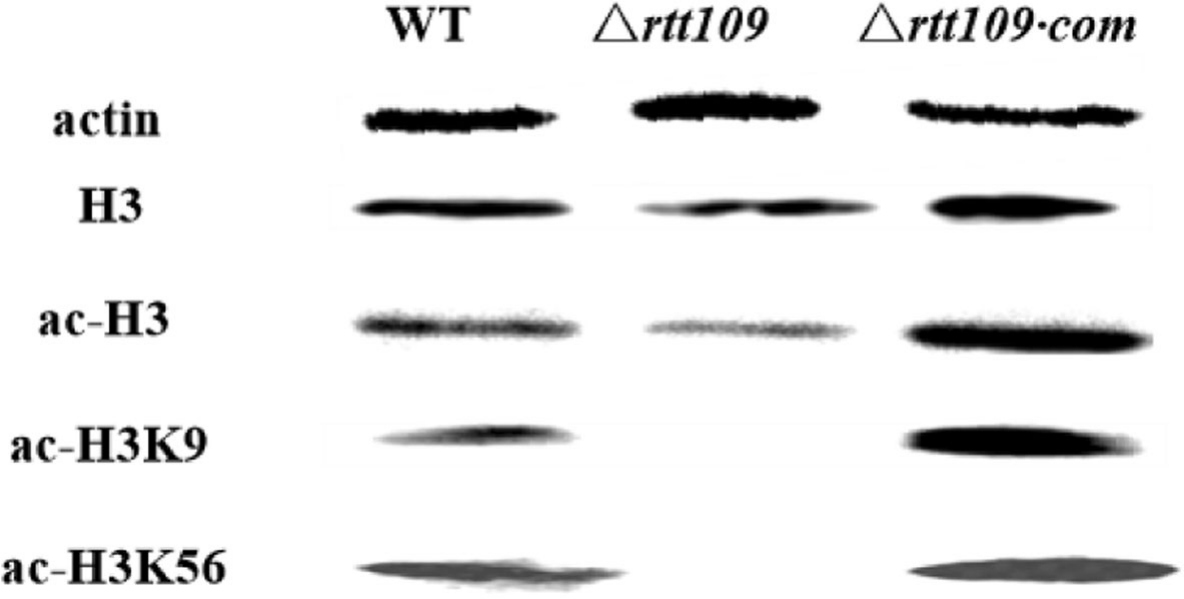
The effect of *rtt109* on the acetylation of histone 3 in *A. flavus*. Western blot analysis showed the acetylation level targeting on H3K9 and H3K56 compared with actin, H3, and ac-H3. Wild type (WT)

In order to confirm our sequence alignment analysis (Fig. 1a), we sought to experimentally confirm that *A. flavus* *rtt109* is a HAT targeting histone 3. As observed, the absence of *rtt109* decreased the H3 acetylation levels, confirming that Rtt109 had HAT activity (Fig. 3). In addition, we examined the acetylation level of lysine residues in histone 3 and found that the acetylation level of Rtt109 targeting H3K9 and H3K56 was largely decreased in Δ*rtt109* compared to WT and △*rtt109*·com strains (Fig. 3). These results confirmed that the *rtt109* was the functional HAT in *A. flavus*.

### Rtt109 played an important role in *A. flavus*’s vegetative growth

**Fig. 4:**
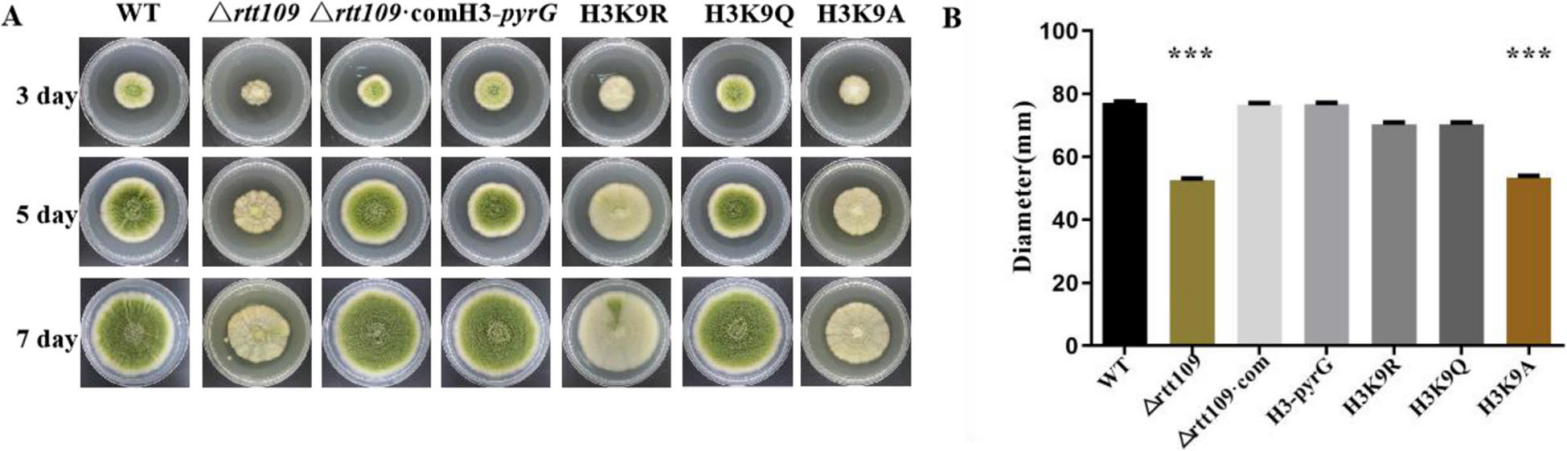
The effect of *rtt109* and H3K9 acetylation on growth in *A.flavus.* **a** The growth of WT, △*rtt109*, △*rtt109*·com, H3-pyrG, H3K9R, H3K9Q, and H3K9A on potato dextrose agar (PDA) solid medium. **b** Statistical analysis of WT, △*rtt109*, △*rtt109*·com, H3-pyrG, H3K9R, H3K9Q, and H3K9A in diameter at day 7

According to the results of Fig. 4, after knocking out *rtt109*, the vegetative growth of *A. flavus* was significantly inhibited. Additionally, the growth rates of the Δ*rtt109* strains were considerably lower than that of the WT and Δ*rtt109*·com strains on PDA and YES media, as well as in YS medium and GMM (Fig. S3B). Microscopic examinations indicated that the Δ*rtt109* strain generated less mycelia and more branches at the mycelial tips compared to the WT and Δ*rtt109*·com strains on YES solid medium (Fig. S3C). These results revealed that *rtt109* played an important role in the vegetative growth in *A. flavus.*

### Rtt109 is essential for *A. flavus*’s conidial formation and Sclerotial generation

**Fig. 5:**
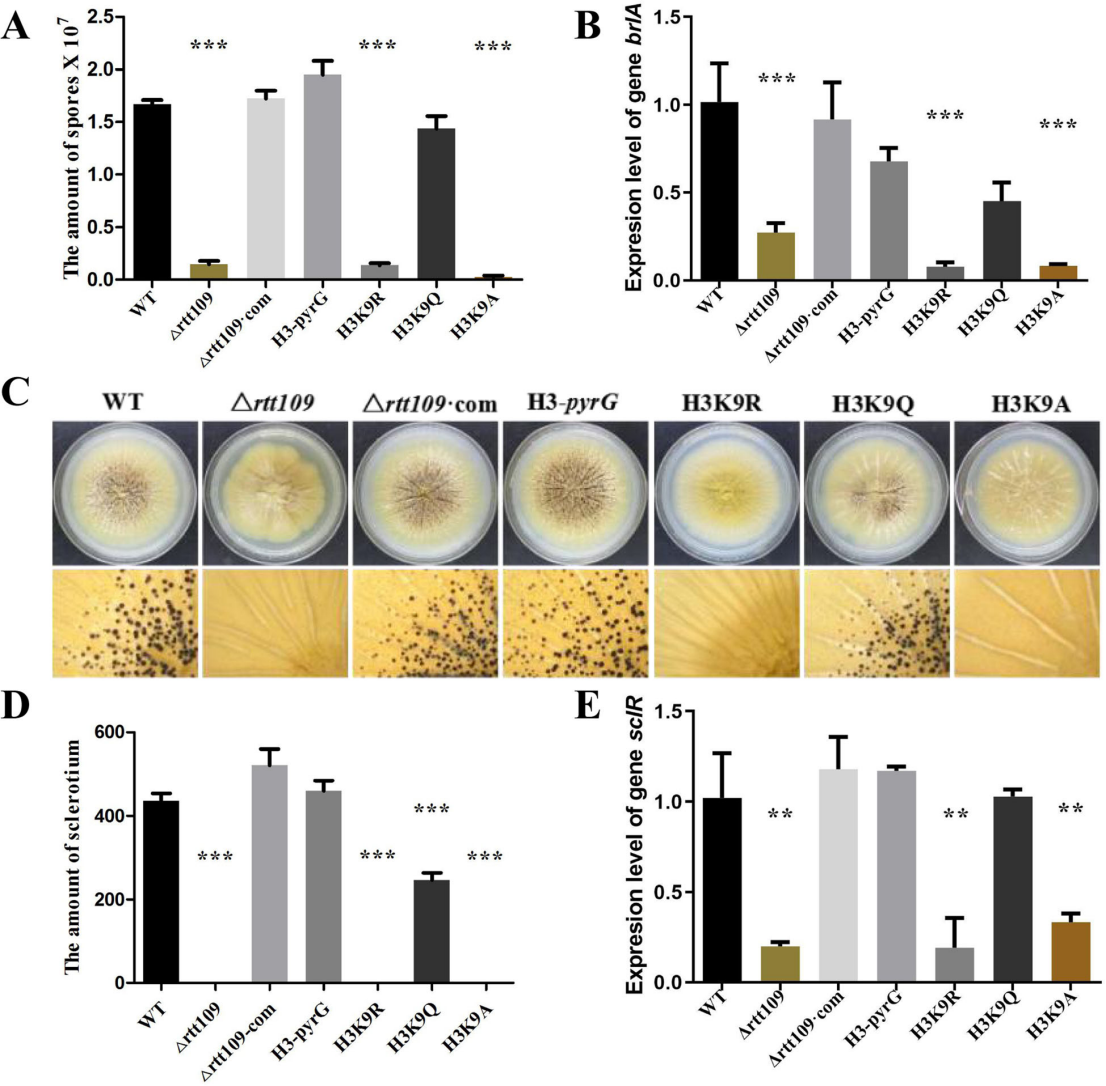
The effect of *rtt109* and H3K9 acetylation on conidia and sclerotia production. WT, △*rtt109*, △*rtt109*·com, H3-pyrG, H3K9R, H3K9Q, and H3K9A strains were used for (**a**) the conidia production on PDA solid medium; (**b**) qRT-PCR analysis of the *brlA* gene; (**c**) sclerotia production analysis under dark conditions at 37 °C; (**d**) statistical analysis; (**e**) qRT-PCR analysis of the *sclR* gene. The asterisks *** represent a significant difference level of *p* < 0.001, and the asterisks ** represent a significant difference level of *p* < 0.01

The conidiophores and conidial formation were analyzed to characterize the role of Δ*rtt109* during reproduction (Fig. 5, S4). Microscopic examinations revealed that the conidial heads of the Δ*rtt109* mutant were smaller than that of the WT and Δ*rtt109*·com strains (Fig. S4A). The quantitative analysis of conidial formation confirmed that the Δ*rtt109* strains was almost unable to form conidia on YES, in contrast to the WT and Δ*rtt109*·com strains (Fig. 5a). Further, the expression of conidial formation related genes, *brlA*, was down-regulated in the Δ*rtt109* strain cultured for 72 h (Fig. 5b). *A. flavus* generated sclerotia, the resting bodies that enable the fungus to survive in unsuitable environments. The WT and Δ*rtt109*·com strain can normally produce sclerotia, while the Δ*rtt109* strain produced none in dark conditions, either on the WKM medium (Fig. S5A) or YS medium (Fig. 5c**)**, and the statistical analysis agreed with this (Fig. 5d**,** S5B). Further, the expression levels of the genes, *sclR,* related to sclerotial production, were lower in Δ*rtt109* compared to the WT and Δ*rtt109*·com strains (Fig. 5e**)**. All these results showed that rtt109 played vital roles in the conidial formation and sclerotial generation in *A. flavus.*

### Rtt109 played an essential role in pathogenicity towards maize

**Fig. 6:**
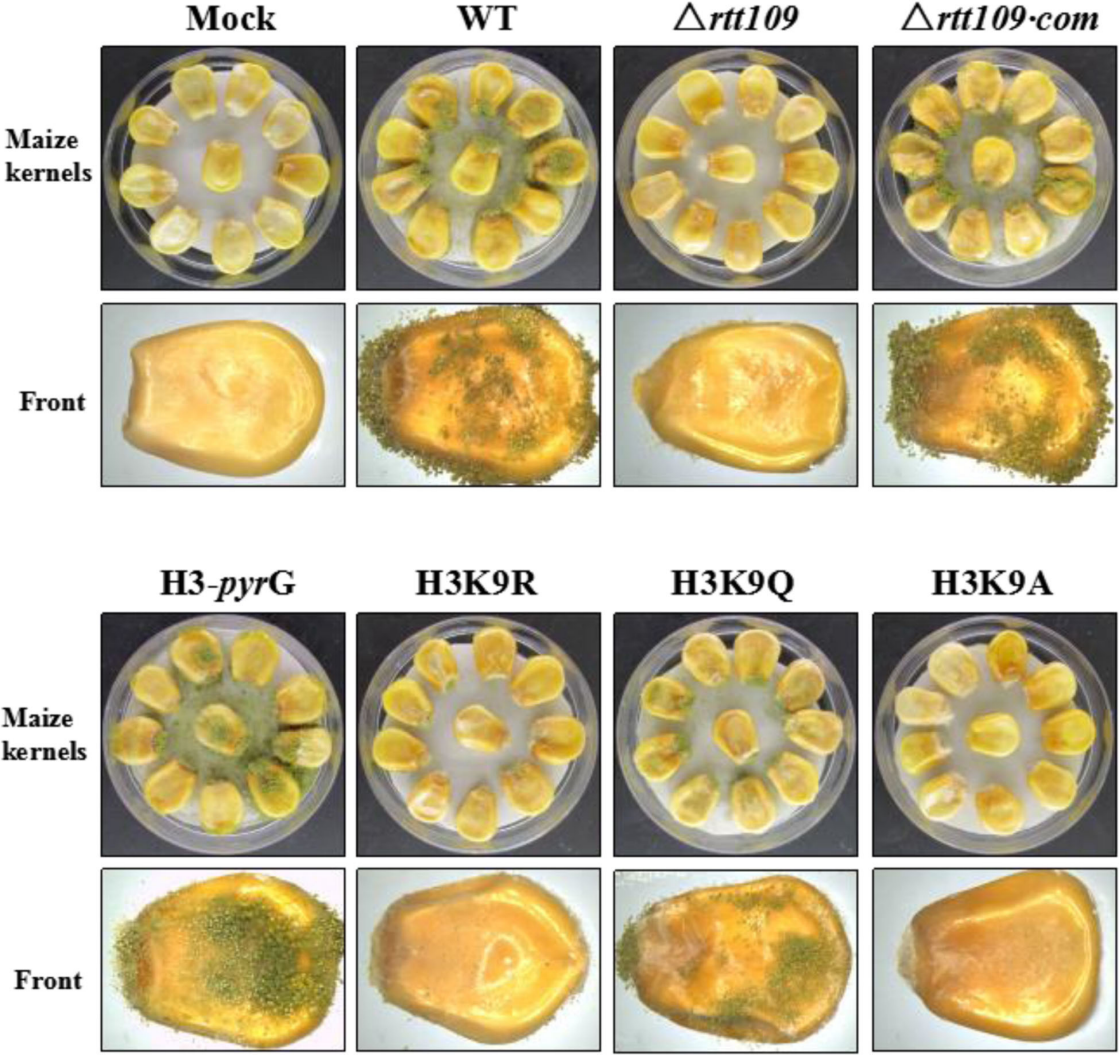
The effect of *rtt109 *and H3K9 acetylation on pathogenicity. The maize infection experiment was carried out in WT, △*rtt109,* △*rtt109*·com, H3-*pyrG*, H3K9R, H3K9Q, and H3K9A strains of *A. flavus* after 7 days in the dark at 29 °C

In order to measure the role that *rtt109* played in affecting maize seed, the ability of all the strains to colonize maize seeds was evaluated. As Fig. 6 shows, the WT, △*rtt109*, and △*rtt109*·com strains were successfully colonized on the maize seed. Among these three strains, the Δ*rtt109* mutant strain was less able to colonize on maize seeds compared to the WT and Δ*rtt109*·com strains. The aerial hyphae of Δ*rtt109* generated on the surface of the colonized maize seeds were much less than those of the WT and Δ*rtt109*·com strains. A lack of conidiation was observed in the Δ*rtt109* mutant-infected maize seeds in contrast to that in the WT and Δ*rtt109*·com strains. In all, the growth of the hyperacetylated strains, WT, △*rtt109*·com, H3-*pyrG* and H3K9Q, are better than the hypoacetylated strains, △*rtt109*, H3K9R and H3K9A. All these results are consisted with the strains grown on mediums and indicated that Rtt109 played an essential role in pathogenicity towards maize.

### Aflatoxin synthesis in *A. flavus* conducted by rtt109

**Fig. 7:**
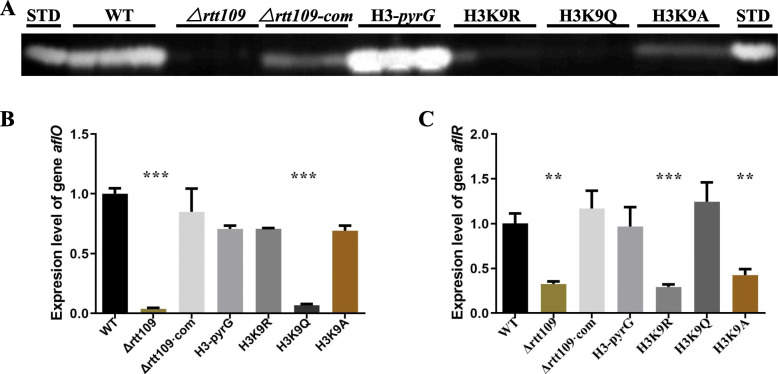
The effect of *rtt109* and H3K9 acetylation on aflatoxin synthesis. **a** TLC assay of aflatoxins produced by the WT, △*rtt109*, △*rtt109*·com, H3-pyrG, H3K9R, H3K9Q, and H3K9A strains grown on YES liquid medium at 29 °C for 3 days. The qRT-PCR analysis results of WT, △*rtt109*, △*rtt109*·com, H3-pyrG, H3K9R, H3K9Q, and H3K9A (**b**) on *aflO*, and (C)on *aflR*. The asterisks *** represent a significant difference level of *p* < 0.001, and the asterisks **represent a significant difference level of *p* < 0.01

In order to investigate the role that *rtt109* played in aflatoxin synthesis, we measured the most important and abundant secondary metabolites aflatoxins in *A. flavus*. The TLC results showed that the aflatoxin productions were largely reduced in Δ*rtt109* (Fig. 7a), indicating that aflatoxin synthesis was affected by *rtt109*. To further analyze the impact on aflatoxin synthesis in *A. flavus* knocking out *rtt109*, qRT-PCR experiments were conducted. In Fig. 7, the expressions of the aflatoxin-specific regulatory genes *aflO* and *aflR* were extremely low in the Δ*rtt109* strain compared to the WT and Δ*rtt109*·com strains (Fig. 7b, c). All these results showed that *rtt109* is important for aflatoxin production.

### Rtt109 is critical for a stress response in *A. flavus*

**Fig. 8:**
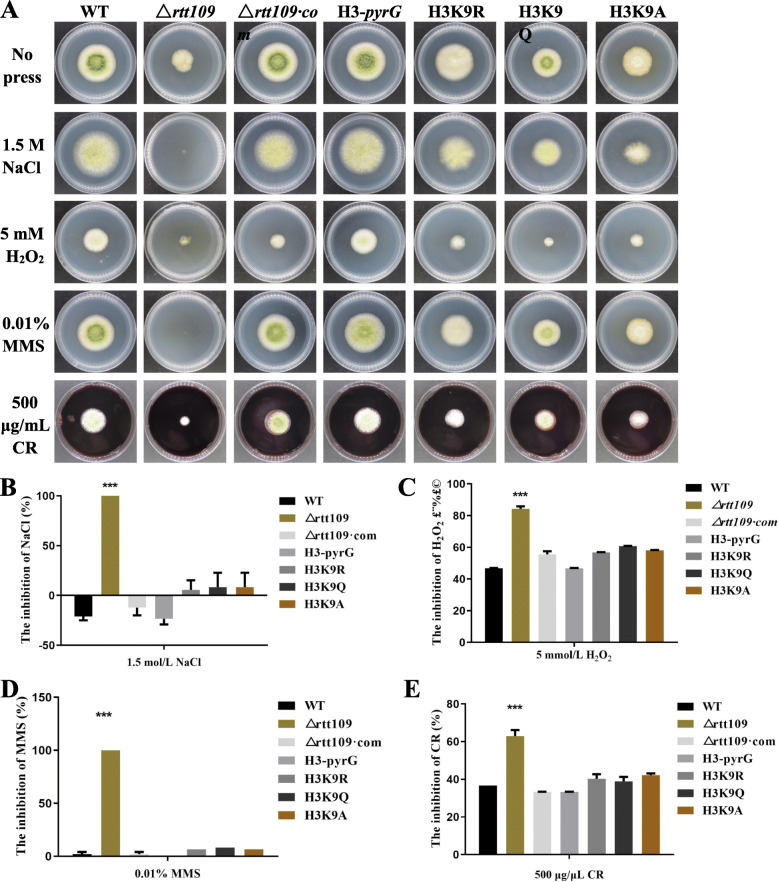
The effect of *rtt109* and H3K9acetylation on stress response. **a** WT, △*rtt109*, △*rtt109*·com, H3-pyrG, H3K9R, H3K9Q and H3K9A strains were cultured on PDA medium containing 0.01% MMS, 5 mol/mL H_2_O_2_, 1.2 mol/L NaCl, and 500 μg/μL Congo red (CR) at 37 °C for 3 days. The inhibition of growth of the strains on PDA medium containing (**B**) 5 mol/mL H_2_O_2_, (**C**) 1.5 mol/L NaCl, (D) 0.01% MMS, (E) 500 μg/μL CR compared with the no press group, respectively. The asterisks *** represent a significant difference level of *p* < 0.001, and the asterisk * represents a significant difference level of *p* < 0.05

We investigated the sensitivity of the WT, Δ*rtt109*, and Δ*rtt109*·com strains to 500 μg/μL CR, 5 mol/mL H_2_O_2_, 0.01% MMS and 1.2 mol/L NaCl, which represent cell wall stress, oxidative stresses, genotoxic stress and osmotic stress, respectively, and find that Δ*rtt109* strain is sensitive to NaCl, H_2_O_2_, MMS and CR. As we can observe in Fig. 8a, the Δ*rtt109* strain could not grow on the PDA medium containing 1.2 mol/L NaCl or 0.01% MMS, and statistical results supported these (Fig. 8b, d). Although Δ*rtt109* strain could grow on the PDA medium containing 500 μg/μL CR and 5 mol/mL H_2_O_2_ respectively, Δ*rtt109* strain was largely inhibited in contrast of WT and Δ*rtt109*·com strains (Fig. 8c, e). These results indicated that Rtt109 played important roles in responsing to cell wall stress, oxidative stresses, osmotic stress and especially the genotoxic stress in *A. flavus*.

### H3K9 as an important Acetylable target for rtt109 in *A. flavus*

In Figs. 4, 5, 6, 7, WT, △*rtt109*·com, H3-pyrG, and the simulative hyperacetylation of H3K9 strain H3K9Q showed similar trends on conidial formation, sclerotial generation, vegetative growth, and pathogenicity towards maize, while △*rtt109*, the simulative hypoacetylation of H3K9 strains H3K9R, and H3K9A was defective in these functions. These results indicated that the acetylation of H3K9 had a great influence on the functions above. However, there was no obvious differences in responding to stress between WT, Δ*rtt109*·com, H3-pyrg, H3K9R, H3K9Q, and H3K9A, which indicated that the stress of *A. flavus* might not be influenced by acetylation of H3K9. All these results confirmed that H3K9 was the acetylated target by rtt109 in *A. flavus,* which played important roles in its full functions.

## Discussion

According to the bioinformatics analysis, the Rtt109 homologous proteins did show an evolutionary trails in fungi, plants and animals, however, the fundamental function of Rtt109 is retained due to the conserved KAT_11 domain (Carrozza et al., 2003). In addition, the characteristic structural domain, KAT_11, is extremely conserved among Rtt109, which is orthologous in fungi, plants, and animals (Carrozza et al., 2003). The results in Fig. 1 might indicate that KAT_11 is an ancient domain and has evolved into different branches. Our results also showed that Rtt109 mainly localized to the vacuole or vesica of *A. flavus*. The previous study of Rtt109 showed that HATs are usually localized in the nucleus and cytoplasm in *S. cerevisiae *(Shahbazian & Grunstein, 2007; Kouzarides, 2000; Torchia et al., 1998; Carrozza et al., 2003; Lee et al., 2000*)*, but sometimes in vacuole (Soukup et al., 2012). It has been reported that Rtt109 of *S. cerevisiae* will be transported out from the vacuole by Vps75, and then delivered to the nucleus in order to activate some functions of the histone. However, there are fundamental differences between *S. cerevisiae* and *A. flavus* in genomics and cell structure, thus the functions and the localization of the HATs Rtt109 are different. For example, in *A. flavus,* there are no similar sequences or protein homologous to Vps75 of *S. cerevisiae.* Therefore, further study on how Rtt109 is delivered from vacuole or vesica of *A. flavus* is necessary.

The previous study in *S. pombe* indicated that Rtt109 can acetylate the lysine residues H3K9, H3K27, and H3K56 of histone 3 (Selth & Svejstrup, 2007a; Han et al., 2007b; Tang et al., 2008; Selth et al., 2009), and we found that Rtt109 was a bona fide HAT for acetylation of histone 3 targeting both H3K9 and H3K56. We also found that the *rtt109* functions for growth, asexual development, sclerotial formation, secondary metabolites synthesis, and invasion towards hosts of *A. flavus* were mediated by H3K9, which indicates that *rtt109* is involved in many physiological functions of *A. flavus* due to its acetyltransferase activity*.* The modification of histones will lead to the expression or silencing of various genes, which is usually conducted by histone modifying enzymes.

When H3K9 was hypoacetylated, growth, sclerotium formation, and infection of *A. flavus* were significantly lower than that from the simulative hyperacetylated H3K9. Simulative hyperacetylation and hypoacetylation of H3K9 both influenced the synthesis of aflatoxin, but the regulation mechanism might be a dynamic process in *A. flavus*. Therefore, Rtt109 is a bona fide HAT for the acetylation of histone 3, and the acetylation modification on H3K9 played an important role in the regulation of the sclerotium formation, secondary metabolism, growth, and other physiological process in *A. flavus*. However, we also found that the hyperacetylation and hypoacetylation were very complex. For example, the expression level of *aflO* in the putative hypoacetylated strains, H3K9R and H3K9A, were consistent with the WT and Δ*rtt109*·com, but Δ*rtt109* and H3K9Q were almost the same. Furthermore, there was no difference in the stress responses between the WT, Δ*rtt109*·com, H3K9R, H3K9Q and H3K9A, showing that the hyperacetylation and hypoacetylation were a complex dynamics process. Therefore, further research on the mechanism of acetylation of H3K9 affecting gene expression is needed.

The importance of Rtt109 for DNA damage repair has been broadly studied in fungal species. Usually, the Rtt109 protein in fungal species is known as a kind of acetyltransferase that can modify H3K56, which is well connected with the expression of *rad52 *(Alvaro et al., 2007*;* Malone et al., 1988), the DNA damage repair function gene in many eukaryote species (Tsubota et al., 2007b; Selth & Svejstrup, 2007a; Han et al., 2007b). However, the importance of *rtt109* for morphogenesis has not yet been well studied in filamentous fungal species. After knocking out the gene *rtt109*, the colony of *A. flavus* was significantly smaller than that of the WT, and branching at the mycelial tips increased significantly. The agent MMS can cause DNA damage of the genome in *A. flavus*, while acetyltransferase Rtt109 can activate the DNA damage repair function of *A. flavus*, thus, the deletion of *rtt109* may lead to the loss of DNA damage repair function.

By microscopic observation, we found that the conidial heads of △*rtt109* were much smaller than those of the WT and △*rtt109*·com, which indicated that development was regulated by gene *rtt109*. As for conidial formation, the amounts of spores produced by △*rtt109* on PDA, YES, YS, and GMM mediums were greatly decreased, in particular, there was no spore generated on the YES medium. The sclerotia production of △*rtt109* on the WKM medium was greatly decreased, in particularly, there was no sclerotia generated on the YS medium. These results indicated that knocking out *rtt109* leads to a defect of reproduction ability in *A. flavus*.

In colonization experiments, we found that △*rtt109* can hardly colonize on the surface of maize, which is consistent with the culture experiments, implying that *rtt109* also played a role in invasion towards the crops. The synthesis of aflatoxin is a complex process in *A. flavus*, which is regulated by a cluster of genes. There were 30 genes reported to participate in aflatoxin synthesis, and the total length of this cluster is 70 kb (Yu et al., 2004; Wild & Turner, 2002; Brown et al., 1996; Chang, 2003). The activation of gene expression is connected to histone modification. According to our results, the △*rtt109* strain showed an inhibition in aflatoxin synthesis, indicating that *rtt109* played an important role in aflatoxin synthesis.

## Conclusion

In this study, our results indicated that rtt109 was involved in regulating many physiological processes in *A. flavus*. After knocking out rtt109, the growth rate, conidium generation, generation of sclerotia, and aflatoxin synthesis of *A. flavus* were largely reduced, indicating that rtt109 is crucial in *A. flavus*. Rtt109 is a bona fide HAT for the acetylation of histone 3, and the acetylation modification on H3K9 played important roles in the regulation of many physiological processes in *A. flavus*.

## Supplementary Information

**Additional file 1: Table S1.** The strains used in this experiment. **Table S2.** PCR primers used in this study. **Table S3.** qRT-PCR primers used in this study. **Fig. S1**. Strategy of construction for *△rtt109* and *△rtt109·com* strains. Diagrammatic representation of the gene replacement strategy for construction of (**A**) △*rtt109*, (**B**) △*rtt109*·com, and (**C**) *rtt109*-mCherry. Sequences A and B referred to the homologous sequences near the target *A. flavus rtt109*. **Fig. S2.** Strategy of construction for point-mutant strains. (A) Diagrammatic representation of the gene replacement strategy for construction of H3 point-mutant strains. (B) Sequence alignment results of H3K9A, H3K9Q, H3K9R, H3-*pyrG*, and WT. **Fig. S3.** The effect of *rtt109* on growth of *A. flavus*. (A) The growth of WT, △*rtt109* and △*rtt109*·com on YES solid medium, YS solid medium and GMM solid medium. (B) Growth rate of WT, △*rtt109* and △*rtt109*·com as in (A). (C) Mycelial branching observation of WT, △*rtt109* and △*rtt109*·com strains grown on YES solid medium. **Fig. S4.** The effect of rtt109 on conidia production in *A. flavus*. (A) The conidophore of WT, △*rtt109* and △*rtt109*·com on YES solid medium, YS solid medium and GMM solid medium. (B) The statistical analysis of conidia production on YES solid medium, YS solid medium and GMM solid medium as in A. The asterisks *** represents a significant different level of *p* < 0.001. **Fig. S5.** The effect of rtt109 on sclerotia production in *A. flavus*. (A) Sclerotia production of the strains cultured on WKM solid medium under light and dark at 37 °C for 10 days. (B) Statistical analysis of sclerotia production in WT, △*rtt109* and △*rtt109*·com grown on WKM solid medium. The asterisks *** represents a significant different level of *p* < 0.001. **Fig. S6.** The effect of rtt109 on aflatoxin production in *A. flavus*. (A) TLC assay of aflatoxin produced by *A. flavus* WT, △rtt109 and △rtt109·com strains grown on PDB liquid medium at 29 °C from 3 to 6 days. (B) HPLC assay of aflatoxin produced by the WT, △*rtt109* and △*rtt109*·com strains grown on YES liquid medium at 29 °C. **Fig. S7.** The effect of strains WT, △*rtt109*, △*rtt109*·com, H3K56R, H3K56Q, and H3K56A on (A) growth on PDA medium, (B) conidophore on GMM medium, (C) sclerotia production.

## Data Availability

Not applicable.
