# Supplementary material for: The Fungi-specific histone Acetyltransferase Rtt109 mediates morphogenesis, Aflatoxin synthesis and pathogenicity in Aspergillus flavus by acetylating H3K9
Source: IMA Fungus. 2021 Apr 7;12:9. doi: 10.1186/s43008-021-00060-4 (PMC8025522; doi:10.1186/s43008-021-00060-4)
Supplement: Supplementary file 1 — Additional file 1: Table S1. The strains used in this experiment. Table S2. PCR primers used in this study. Table S3. qRT-PCR primers used in this study. Fig. S1. Strategy of construction for △rtt109 and △rtt109·com strains. Diagrammatic representation of the gene replacement strategy for construction of (A) △rtt109, (B) △rtt109·com, and (C) rtt109-mCherry. Sequences A and B referred to the homologous sequences near the target A. flavus rtt109. Fig. S2. Strategy of construction for point-mutant strains. (A) Diagrammatic representation of the gene replacement strategy for construction of H3 point-mutant strains. (B) Sequence alignment results of H3K9A, H3K9Q, H3K9R, H3-pyrG, and WT. Fig. S3. The effect of rtt109 on growth of A. flavus. (A) The growth of WT, △rtt109 and △rtt109·com on YES solid medium, YS solid medium and GMM solid medium. (B) Growth rate of WT, △rtt109 and △rtt109·com as in (A). (C) Mycelial branching observation of WT, △rtt109 and △rtt109·com strains grown on YES solid medium. Fig. S4. The effect of rtt109 on conidia production in A. flavus. (A) The conidophore of WT, △rtt109 and △rtt109·com on YES solid medium, YS solid medium and GMM solid medium. (B) The statistical analysis of conidia production on YES solid medium, YS solid medium and GMM solid medium as in A. The asterisks *** represents a significant different level of p < 0.001. Fig. S5. The effect of rtt109 on sclerotia production in A. flavus. (A) Sclerotia production of the strains cultured on WKM solid medium under light and dark at 37 °C for 10 days. (B) Statistical analysis of sclerotia production in WT, △rtt109 and △rtt109·com grown on WKM solid medium. The asterisks *** represents a significant different level of p < 0.001. Fig. S6. The effect of rtt109 on aflatoxin production in A. flavus. (A) TLC assay of aflatoxin produced by A. flavus WT, △rtt109 and △rtt109·com strains grown on PDB liquid medium at 29 °C from 3 to 6 days. (B) HPLC assay of aflatoxin produced by the WT, △rtt109 a [file 43008_2021_60_MOESM1_ESM.docx]

**Supplementary Materials:**

**Table S1** The strains used in this experiment.

| Strain | Description | Source |
| --- | --- | --- |
| Aspergillus flavus *A. flavus PTS∆pyrG* | ku70-，*pyrG*- | Granted by Dr. Perng Kuang Chang |
| Wild type | ku70- | Constructed in this laboratory |
| △*rtt109* | ku70-，*pyrG*-，△Af*rtt109*::*pyrG* | In this study |
| △*rtt109*·com | ku70-，*pyrG*-，△Af*rtt109*::*pyrG*-ptrA | In this study |
| *rtt109*-mCherry | ku70-，*pyrG*-，△Af*rtt109*-mCherry-*pyrG* | In this study |
| H3-*pyrG* | ku70-，*pyrG*-，AfH3:: *pyrG* | In this study |
| H3K9R | ku70-，*pyrG*-，H3K9R:: *pyrG* | In this study |
| H3K9Q | ku70-，*pyrG*-，H3K9Q:: *pyrG* | In this study |
| H3K9A | ku70-，*pyrG*-，H3K9A:: *pyrG* | In this study |

**Table S2** PCR primers used in this study

| Primer | Sequence（5’-3’） | Characteristics | Source |
| --- | --- | --- | --- |
| *rtt109*-NF | CATTGCACCAGTGGCGTCTT | To construct △*rtt109* | In this study |
| *rtt109*-NR | GGTCAGTTTGTCGGCAGGAT | To construct △*rtt109* | In this study |
| *rtt109*-AF | CTCCCACAAGTAGGCCACA | To construct △*rtt109* | In this study |
| *rtt109*-AR | GGGTGAAGAGCATTGTTTGAGGCCCAGAAGCCTCACGGATG | To construct △*rtt109* | In this study |
| *rtt109*-BF | GCATCAGTGCCTCCTCTCAGACTAGCAGATGCCTTTCGC | To construct △*rtt109* | In this study |
| *rtt109*-BR | TTTGGTCCCAGGTCGTA | To construct △*rtt109* | In this study |
| *rtt109*-OF | AAGGTCACCATTCGCCATAT | To identify △*rtt109* | In this study |
| *rtt109*-OR | ATCCTACCAGTCGTCCAGCA | To identify △*rtt109* | In this study |
| *rtt109*-C-NF | CAGGTCGGGCACGATGAA | To construct △*rtt109*·com | In this study |
| *rtt109*-C-NR | GGTCGGGAGCAGCGTAGAT | To construct △*rtt109*·com | In this study |
| *rtt109*-C-AF | AGATACCAGTCCACTCCCACA | To construct △*rtt109*·com | In this study |
| *rtt109*-C-AR | ACCAATAGACCGAAATCGGAAGAACCCAAATCGCTGA | To construct △*rtt109*·com | In this study |
| ptrA-F | CGATTTCGGTCTATTGGT | To amplify ptrA sequance | [1] |
| ptrA-R | GGGTGAAGAGCATTGTTTGAGGCCGACACGGAAATGTTGAA | To amplify ptrA sequance | [1] |
| *rtt109*-mCherry-AR | CTCGCCCTTGCTCACCATGGCCTTCTTCTTCTTCCGGATCA | To construct *rtt109*-mCherry sequances A | In this study |
| *rtt109*-mCherry-BF | GCATCAGTGCCTCCTCTCAGACTCGGGATTATGGGACA | To construct *rtt109*-mCherry sequances B | In this study |
| *rtt109*-mCherry-BR | GAGGCAGTTGCGGAGT | To construct *rtt109*-mCherry sequances B | In this study |
| *rtt109*-mCherry-NF | TGATCAAATGGTGGTGTCGC | To construct *rtt109*-mCherry sequances | In this study |
| *rtt109*-mCherry-NR | CAGTTTGTCGGCAGGATAGTC | To construct *rtt109*-mCherry sequances | In this study |
| *pyrG*-F | GCCTCAAACAATGCTCTTCACCC | To construct *pyrG* sequances | [2] |
| *pyrG*-R | GTCTGAGAGGAGGCACTGATGC | To construct *pyrG* sequances | [2] |
| H3K9-A1F | CCGAAATCACGGCTTATG | To construct H3K9R sequances A1 | In this study |
| H3K9R-A1 | CCTTGCCACCAGTGGACT | To construct H3K9R sequances A1 | In this study |
| H3K9Q-A1 | CCTTGCCACCAGTGGATG | To construct H3K9Q sequances A1 | In this study |
| H3K9A-A1 | CCTTGCCACCAGTGGAGC | To construct H3K9A sequances A1 | In this study |
| H3K9R-A2F | ACATCCATCCAGGTAGATCCAC | To construct H3K9R sequances A2 | In this study |
| H3K9Q-A2F | ACATCCATCCAGGTCAATCCAC | To construct H3K9Q sequances A2 | In this study |
| H3K9A-A2F | ACATCCATCCAGGTGCGTCCAC | To construct H3K9A sequances A2 | In this study |
| H3K9-A2R | GGGTGAAGAGCATTGTTTGAGGCAGTGCCGAAACCCATTGATA | To construct H3K9 sequances A2 | In this study |
| H3K9-BF | GCATCAGTGCCTCCTCTCAGACGGGATTAGTTTTCGTTTTGC | To construct H3K9 sequances B | In this study |
| H3K9-BR | CGGGTCCTTGTTCTTTTGTTA | To construct H3K9 sequances B | In this study |
| H3K9-NF | CAGATCACCACCCACCCTT | To construct H3K9 sequances | In this study |
| H3K9-NR | TGGGCAGTACACCAGCAAC | To construct H3K9 sequances | In this study |
| H3-F | CTTCGCTTACTACAAATACCTTC | For sequencing | In this study |
| H3-R | CCTAATCGCCTACCCAGA | For sequencing | In this study |
| P801 | CAGGAGTTCTCGGGTTGTCG | To detect *prrG* | [1] |
| P1020 | ATCGGCAATACCGTCCAGGAGC | To detect *prrG* | [1] |
| mCherry-F | ATGGTGAGCAAGGGCGAG | To construct mCherry sequences | [2] |
| mCherry-R | GGGTGAAGAGCATTGTTTGAGGCCTACTTGTACAGCTCGTCCAT | To construct mCherry sequences | [2] |

**Table S3** qRT-PCR primers used in this study

| Primer | Sequence（5’-3’） | Characteristics | Source |
| --- | --- | --- | --- |
| *β-actin-*QF | ACGGTGTCGTCACAAACTGG | To detect *β-actin* | [1] |
| *β-actin-*QR | CGGTTGGACTTAGGGTTGATAG | To detect *β-actin* | [1] |
| *aflO*-QF | GATTGGGATGTGGTCATGCGATT | To detect *aflO* | [1] |
| *aflO*-QR | GCCTGGGTCCGAAGAATGC | To detect *aflO* | [1] |
| *aflR*-QF | AAAGCACCCTGTCTTCCCTAAC | To detect *aflR* | [1] |
| *aflR*-QR | GAAGAGGTGGGTCAGTGTTTGTAG | To detect *aflR* | [1] |
| *scl*R-QF | CAATGAGCCTATGGGAGTGG | To detect *scl*R | [1] |
| *scl*R-QR | ATCTTCGCCCGAGTGGTT | To detect *scl*R | [1] |
| *rtt109*-flu1-F | CTGCTGGACGACTGGTAGGA | To detect *rtt109* | In this study |
| *rtt109*-flu1-R | GGACGGTGGTTGTCAAGGAA | To detect *rtt109* | In this study |
| *rtt109*-flu2-F | TCAAGGTCACCATTCGCCATAT | To detect *rtt109* | In this study |
| *rtt109*-flu2-R | TGCTCGTCGGCATTCACG | To detect *rtt109* | In this study |
| *brl*A-QF | GCCTCCAGCGTCAACCTTC | To detect *brl*A | [1] |
| *brl*A-QR | TCTCTTCAAATGCTCTTGCCTC | To detect *brl*A | [1] |


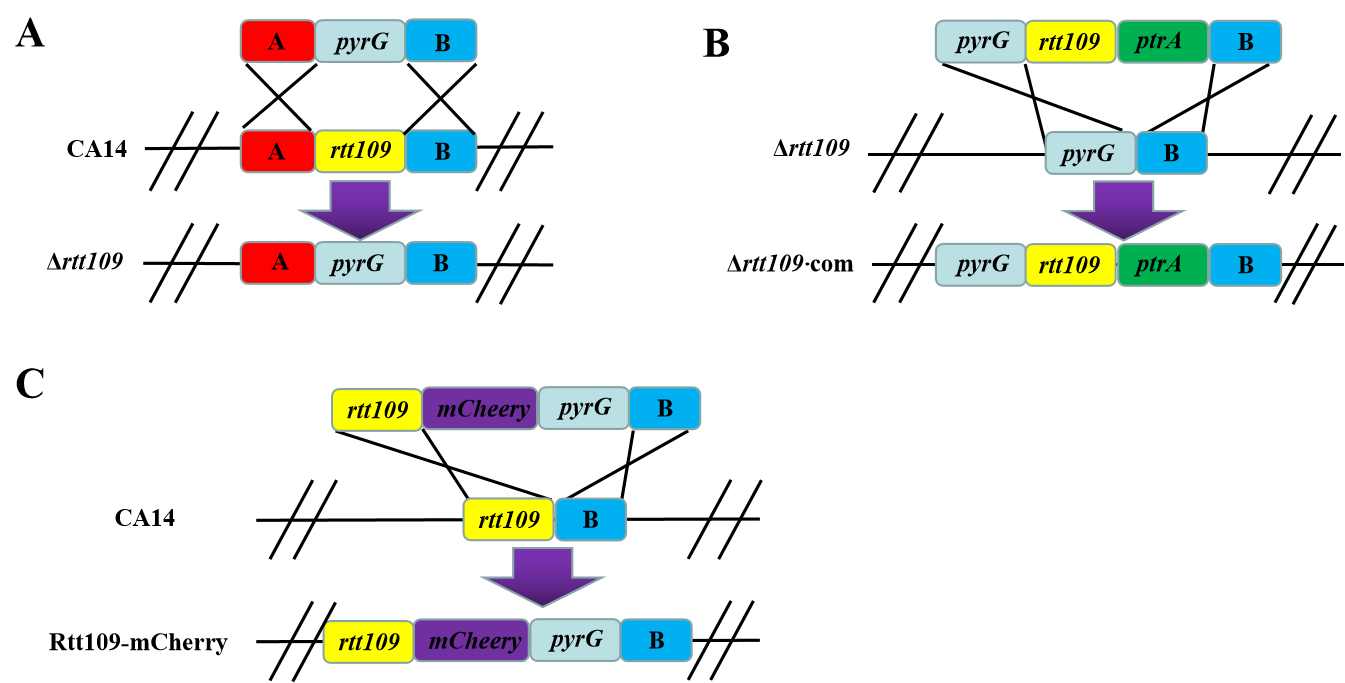


**Figure S1**. Strategy of construction for *△rtt109* and *△rtt109·com* strains. Diagrammatic representation of the gene replacement strategy for construction of (**A**) △*rtt109*, (**B**) △*rtt109*·com, and (**C**) Rtt109-mCherry. Sequences A and B referred to the homologous sequences near the target *A. flavus* *rtt109*.


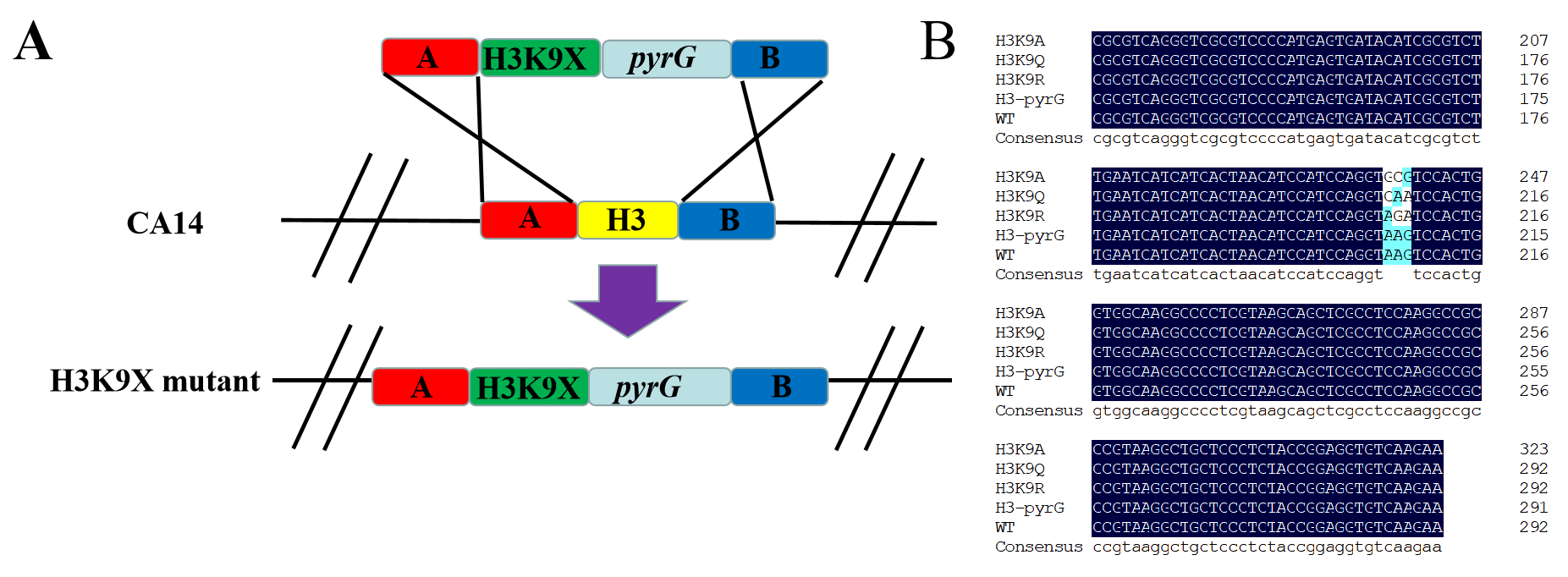


**Figure S2**. Strategy of construction for point-mutant strains. (A) Diagrammatic representation of the gene replacement strategy for construction of H3 point-mutant strains. (B) Sequence alignment results of H3K9A, H3K9Q, H3K9R, H3-*pyrG*, and WT.


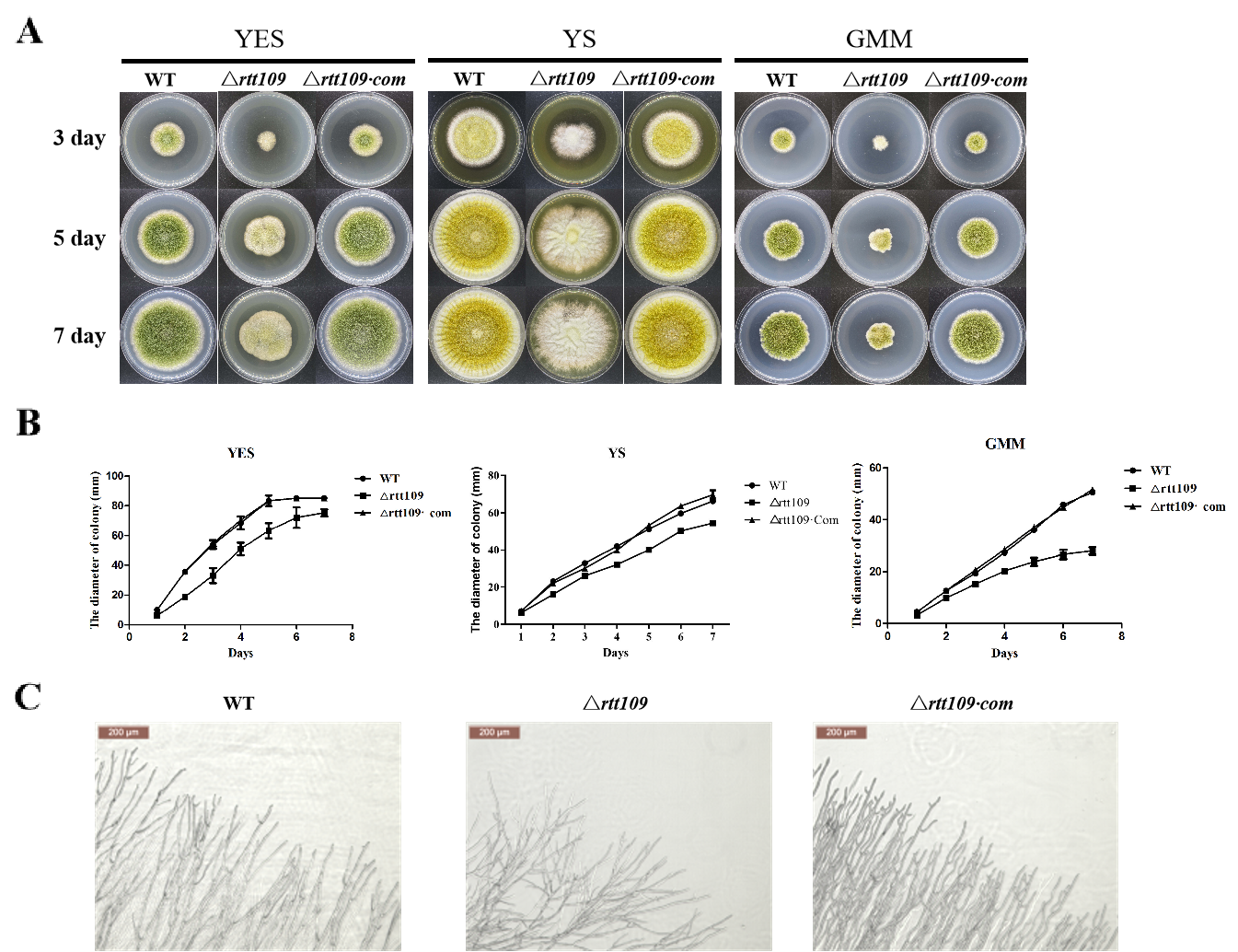


**Figure S3**. The effect of *rtt109* on growth of *A. flavus*. (A) The growth of WT, △*rtt109* and △*rtt109*·com on YES solid medium, YS solid medium and GMM solid medium. (B) Growth rate of WT, △*rtt109* and △*rtt109*·com as in (A). (C) Mycelial branching observation of WT, △*rtt109* and △*rtt109*·com strains grown on YES solid medium.


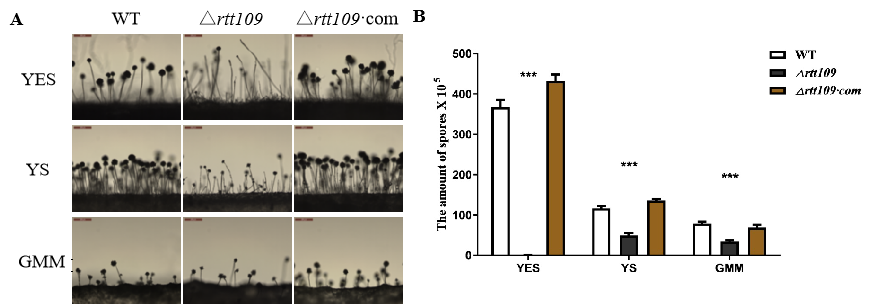


**Figure S4**. The effect of rtt109 on conidia production in A. flavus. (A) The conidophore of WT, △*rtt109* and △*rtt109*·com on YES solid medium, YS solid medium and GMM solid medium. (B) The statistical analysis of conidia production on YES solid medium, YS solid medium and GMM solid medium as in A. The asterisks *** represents a significant different level of p<0.001.


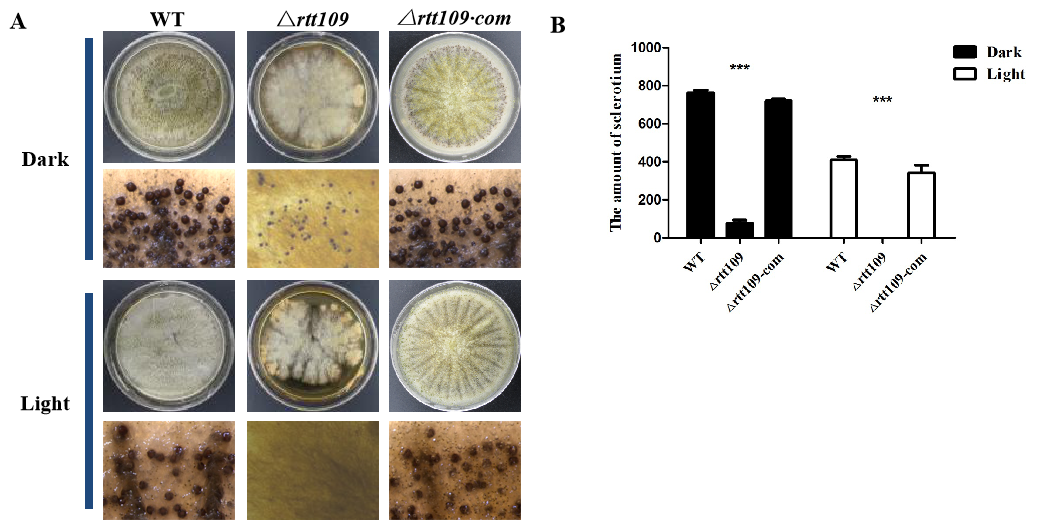


**Figure S5**. The effect of rtt109 on sclerotia production in *A. flavus*. (A) Sclerotia production of the strains cultured on WKM solid medium under light and dark at 37℃ for 10 days. (B) Statistical analysis of sclerotia production in WT, △rtt109 and △rtt109·com grown on WKM solid medium. The asterisks *** represents a significant different level of p<0.001.


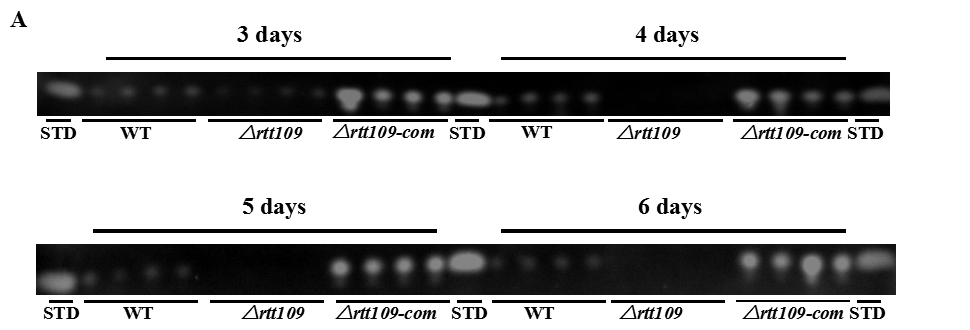

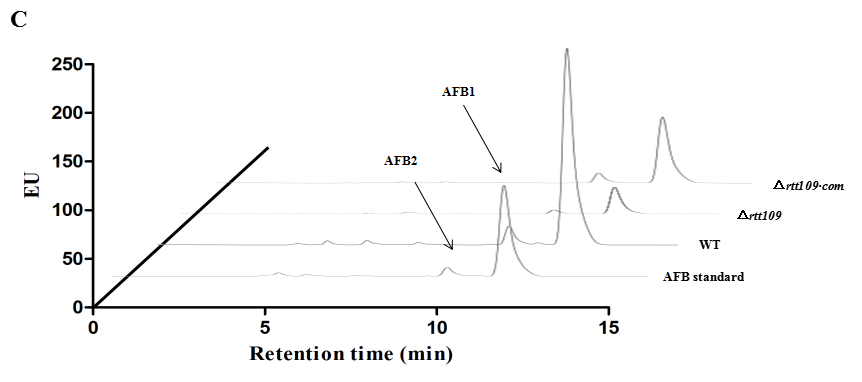

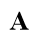

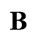


**Figure S6**. The effect of rtt109 on aflatoxin production in *A. flavus*. (A) TLC assay of aflatoxin produced by *A. flavus* WT, △rtt109 and △rtt109·com strains grown on PDB liquid medium at 29℃ from 3 to 6 days. (B) HPLC assay of aflatoxin produced by the WT, △rtt109 and △rtt109·com strains grown on YES liquid medium at 29℃.


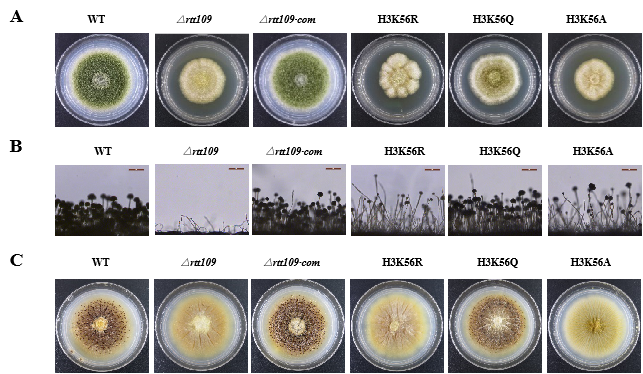


**Figure S7**. The effect of strains WT, △*rtt109*, △*rtt109*·com, H3K56R, H3K56Q, and H3K56A on (A) growth on PDA medium, (B) conidophore on GMM medium, (C) sclerotia production.

**References**

1. Lan H, Sun R, Fan K, et al. The Aspergillus flavus histone acetyltransferase AflGcnE regulates morphogenesis, aflatoxin biosynthesis, and pathogenicity[J]. Frontiers in microbiology, 2016, 7: 1324.
2. Wong K H, Todd R B, Oakley B R, et al. Sumoylation in Aspergillus nidulans: sumO inactivation, overexpression and live-cell imaging[J]. Fungal Genetics and Biology, 2008, 45(5): 728-737.
